# Role of Transposable Elements in Genome Stability: Implications for Health and Disease

**DOI:** 10.3390/ijms23147802

**Published:** 2022-07-15

**Authors:** Audesh Bhat, Trupti Ghatage, Sonali Bhan, Ganesh P. Lahane, Arti Dhar, Rakesh Kumar, Raj K. Pandita, Krishna M. Bhat, Kenneth S. Ramos, Tej K. Pandita

**Affiliations:** 1 Centre for Molecular Biology, Central University of Jammu, Jammu 181143, India; sonalibhan62@gmail.com; 2 Department of Pharmacy, BITS-Pilani Hyderabad Campus, Hyderabad 500078, India; p20190450@hyderabad.bits-pilani.ac.in (T.G.); p20200470@hyderabad.bits-pilani.ac.in (G.P.L.); artidhar@hyderabad.bits-pilani.ac.in (A.D.); 3 Department of Biotechnology, Shri Mata Vaishnav Devi University, Katra 182320, India; kumar.rakesh@smvdu.ac.in; 4 Baylor College of Medicine, One Baylor Plaza, Houston, TX 77030, USA; raj.pandita@bcm.edu; 5 Department of Molecular Medicine, University of South Florida, Tampa, FL 33612, USA; kmbhats18@gmail.com; 6 Center for Genomics and Precision Medicine, Texas A&M College of Medicine, Houston, TX 77030, USA; kramos@tamu.edu

**Keywords:** transposons, DSB, genome, stability

## Abstract

Most living organisms have in their genome a sizable proportion of DNA sequences capable of mobilization; these sequences are commonly referred to as transposons, transposable elements (TEs), or jumping genes. Although long thought to have no biological significance, advances in DNA sequencing and analytical technologies have enabled precise characterization of TEs and confirmed their ubiquitous presence across all forms of life. These findings have ignited intense debates over their biological significance. The available evidence now supports the notion that TEs exert major influence over many biological aspects of organismal life. Transposable elements contribute significantly to the evolution of the genome by giving rise to genetic variations in both active and passive modes. Due to their intrinsic nature of mobility within the genome, TEs primarily cause gene disruption and large-scale genomic alterations including inversions, deletions, and duplications. Besides genomic instability, growing evidence also points to many physiologically important functions of TEs, such as gene regulation through cis-acting control elements and modulation of the transcriptome through epigenetic control. In this review, we discuss the latest evidence demonstrating the impact of TEs on genome stability and the underling mechanisms, including those developed to mitigate the deleterious impact of TEs on genomic stability and human health. We have also highlighted the potential therapeutic application of TEs.

## 1. Introduction

**Figure 1:**
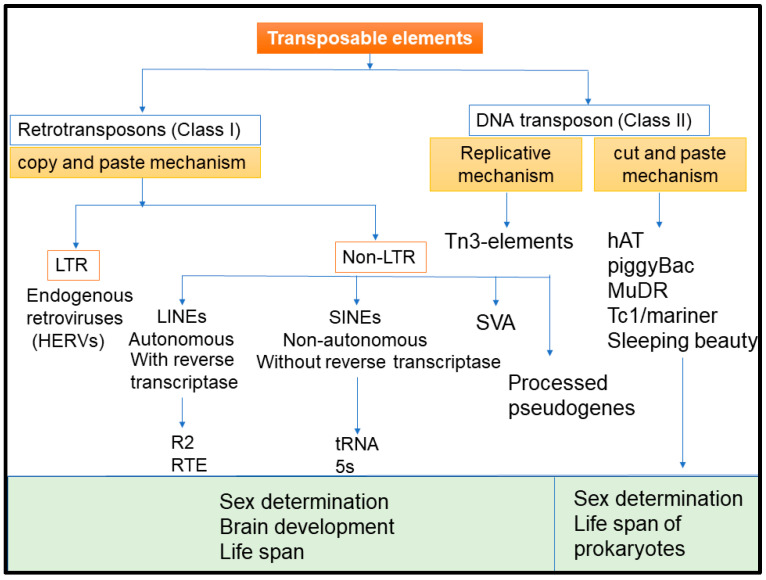
Classification of human transposons.

Transposable elements (TEs) were first discovered by Barbara McClintock as “jumping genes” in maize in the late 1940s [1]. Subsequently, TEs were characterized as ubiquitously present mobile DNA sequences in plants and animals that integrate into new sites within the genome following mobilization, often leading to duplications, insertions, deletions, and/or creation or reversion of mutations which in turn lead to altered cellular genotypes. The mammalian genome is comprised of about 45% TEs that are categorized as DNA transposons and retrotransposons [2]. DNA transposons, also referred as Class II TEs, comprise about 3% of our genome [3] but are less-characterized due to their lesser biological significance as compared to the retrotransposons, also referred as Class I TEs. The DNA transposons predominantly transpose via a cut-and-paste mechanism, also known as a conservative or non-replicative mechanism, by encoding a transposase required for the transposition or via a less commonly used mechanism known as replicative transposition [4]. During replicative transposition, the transposons replicate as a part of the chromosome, followed by transposition of the newly replicated transposon to a locus in front of the replication fork. DNA transposons consist of two terminal inverted repeats (TIRs) that flank a transposase gene body. These TIRs are recognized by transposases to perform transposon DNA excision followed by insertion of the excised DNA into a new genomic location [4]. A unique hallmark associated with DNA transposons is the target site duplication (TSD) event that happens after the insertion at new location is completed [4]. Out of the nine superfamilies of DNA transposons identified in the eukaryotic genome, the human genome possesses eight superfamilies viz *hAT*, *Tc1*/*mariner*, *Crypton*, *Helitron*, *Kolobok*, *Merlin*, *MuDR*, and *piggyBac*, with *hAT* and *Tc1/mariner* superfamilies being the predominant ones [5,6]. Retrotransposons, which have been subjected to extensive investigation, as compared to the DNA transposons, move within the genome via a copy-and-paste mechanism by forming RNA intermediates which are then reverse-transcribed to DNA, followed by their integration at new genomic sites [7]. Retrotransposons are further classified into two classes: long terminal repeat (LTR) and non-LTR transposons, based on the presence or absence of long terminal repeats (Figure 1). In humans, LTR transposons include human endogenous retroviruses (HERVs), which are estimated to have been incorporated into the genome more than 25 million years ago [2]. Human endogenous retroviruses constitute 8% of the genome, most of which is either in the form of single LTRs (2%) or defective mammalian-apparent LTR retrotransposon (MaLR) elements (4%) [3]. Like their exogenous retrovirus counterparts, the majority of the HERVs have a typical proviral structure of two LTRs that flank the viral *gag*, *pro-pol*, and *env* genes, with a few HERVs, such as HERV-K, having a more complex genome. The non-LTR transposons include Long Interspersed Nuclear Elements (LINEs) and Short Interspersed Nuclear Elements (SINEs), among which LINE-1, Alu, SVA (SINE, VNTR and Alu) elements are the only ones currently active in human genome [8,9,10,11]. Of note, LINE-1s are the only autonomously active family of retrotransposons present in the human genome [12].

Although it was originally thought that TEs are devoid of any biological role, technological advances over the past 20 years have enabled greater understanding and appreciation of their roles in gene regulation and expression [8,9,10,11]. Transposon elements also contribute to genomic evolution and are thus considered useful markers of plasticity [13]. However, mobilization of TEs often poses a threat to genome integrity as they can be linked to silent mutations, non-allelic homologous recombination (NAHR), alternative splicing, and various epigenetic changes leading to genomic instability [14]. The genomic instability caused by TE insertions can accelerate various diseases, including genetic disorders, psychiatric disorders, and cancer [15,16,17,18,19,20]. Though transcriptional and posttranscriptional mechanisms have evolved to neutralize most TE insertions in the host cell [21], several diseases have been linked to more than 120 TE insertions [22]. DNA sequencing technology has enabled the scientific community to precisely characterize the location of and polymorphisms associated with TEs [23]. Also, computational tools have added to our understanding as to how epigenetic changes caused by TE insertions can have a significant effect on the expression of neighboring genes [23,24]. The activity of TEs is not only restricted to deleterious impacts but also involves the generation of regulatory differences in the human genome [25]. In this review, we discuss the impact of TEs on genomic stability, their physiological functions in the host cell, the role of TEs in human diseases, and their therapeutic potential.

## 2. Transposable Element Induced Genomic/Epigenomic Instability and Tumorogenesis

The characteristic features of TEs include their ubiquitous presence as repetitive elements along with their ability to mobilize within the genome. Due to these properties, both Class I and Class II TEs serve as frequent causal factors in genome rearrangements. Genomic alterations involving TE insertions are often deleterious in nature and range from insertions/deletions to large-scale chromosomal rearrangements [26]. Activated TEs have the potential to enter alternate genomic sites to disrupt the DNA repair process, thereby inducing genomic instability in the host cell. DNA transposons, for instance, inactivate or dysregulate gene expression by insertion within intronic, exonic, or other regulatory regions [27,28]. PiggyBac transposable element derived 5 (*PGBD5*) gene, belonging to the class of piggyBac TEs, was shown to be an oncogenic mutator by causing site specific DNA rearrangements, thus leading to various aggressive childhood tumors [29]. In another instance, the recombination-activating gene 1 and 2 (RAG1/2) recombinases, despite playing an essential role in V(D)J recombination in developing lymphocytes, also exhibit high similarity to DNA transposons by promoting chromosomal translocations and deletions, thereby inducing lymphocytic malignancies [30]. Additionally, the human THAP domain containing 9 (THAP9) gene is found to encode an active DNA transposase responsible for transposition of P elements across the species [31]. Endonucleases encoded by the autonomous LINE-1 retrotransposons are regarded as the main culprits of DNA double-stranded breaks (DSBs), thus disrupting cellular DNA repair systems [26,32]. LINE-1 integration into the genome can also lead to large deletions and rearrangements of the genomic sequence, which in turn halt and modulate the DNA repair process [33,34]. The impact of retrotransposon insertions has been well-studied, with about 0.3% of total mutations estimated to be caused by retrotransposon insertions [35]. Insertion of TEs into coding regions of the genome may lead to frameshift mutations associated with premature termination or may induce missense or non-sense mutations. For instance, insertion of Alu elements into exonic regions of mRNA causes a change in the open reading frame (ORF) of that particular coding region and affects gene expression [35]. Also, insertion of TEs into intronic regions can have deleterious effects, as insertion of Alu elements and LINE-1 within an intron can introduce novel splice sites, leading to alternative splicing events that disrupt transcriptional integrity [36,37]. Some studies have also shown that insertion of TEs into 5′ or 3′ regions of genes may disrupt normal gene expression [38]. Therefore, the collective impact of alterations in gene expression due to TE insertions has been associated with various disease conditions such as cancer and genetic disorders [39].

It has been suggested that the presence of TE sequences, even without the ability to transpose, can disrupt the integrity of the genome [40]. The insertion of TEs often attracts epigenetic modifiers that not only modify the transposon sequences but also impact surrounding regions. For instance, the suppression of CpG rich TE inserts by methylation eventually leads to the loss of CpG sites in the surrounding DNA of the host genome [41]. Also, it has been reported that short repeats tend to be the hotspots of genomic instability, as TE sequences are prominent sites for DSBs in cancer cell genomes [42]. Short, inverted repeats like Alu elements are believed to form hairpin structures which cause stalling of DNA replication [43]. Also, novel inverted repeats may be created by the insertion of de novo Alu elements, resulting in genetic rearrangements. Due to their ubiquitous presence throughout the genome, TEs have been associated with NAHR translocations. Some studies have revealed that Alu-mediated NAHR have had high impacts on genomic deletions, surpassing those associated with LINE-1 mediated NAHR [44,45].

The methylation status of DNA is thought to directly correspond to disease states like cancer [46]. Due to global hypomethylation and epigenetic dysfunction in cancer cells, non-LTR retrotransposon activation is most prominently observed during oncogenesis [39,47]. Insertion of Alu elements into DNA repair genes such as breast cancer 1 genes (*BRCA1*) and 2 (*BRCA2*) [19,48], LINE-1 retrotransposon into tumor suppressor gene adenomatous polyposis coli (*APC*) [20], and retinoblastoma 1 (*RB1*) gene [49] cause disruption in the function of these genes, thus leading to tumorogenesis. It is now well-documented that active retrotransposon like Alu and LINE-1 elements are associated with tumorigenesis [50,51,52,53]. For instance, promoters of LINE-1 element are found to be demethylated in cancer cells [54]. Promoter demethylation often leads to gene activation and alterations in global gene expression [55,56]. Conversely, methylation of TE promoters renders them inactive and is believed to be a primary mechanism for suppression of transposition in somatic cells [57].

In the eutherian genome, a unique case has been described in which envelope glycoprotein-encoding (*env*) genes of endogenous retroviruses have been adopted by the mammalian genome and converted into a functional gene that expresses syncytin-1 and -2 proteins, important contributors to normal placental architecture and trophoblast turnover [58]. Syncytin-1 can also induce invasion and metastasis through endothelial to mesenchymal transition in endometrial carcinoma [59] and serves as a positive indicator of recurrence-free survival in breast cancer patients [60]. Various cancers, such as cutaneous T-cell lymphomas and leukemias, express syncytin-1 [61]. The expression of syncytin-1 has been associated with a decrease in average survival in rectal but not colon cancer patients, suggesting that syncytin-1 can be site-specific [62]. Likewise, upregulation of syncytin-1 activates the epithelial-to-mesenchymal transition (EMT) pathway in endometrial carcinoma patients. Hence, ERV TEs specifically alter tumor growth by acting as promoter enhancers at the protein level [63]. Endogenous retroviruses have oncogenic potential, as evidenced by the finding that increased ERV-K expression in healthy mice disrupts germ cell maturation, resulting in the development of seminomas. However, knockdown of ERVs through shRNA/siRNA decreases the tumor growth in mouse xenograft and cell-line models [64,65].

The impact of post-insertion events of TEs is global and influences genome structure, chromosome dynamics, and function. Transposon elements inserted into untranslated regions like introns, downstream, and upstream might act as enhancers and promoters for the target genes [66]. Post-insertion events from NAHR contribute to deletions, segmental duplicates, and inversions. An altered expression of self-propagating LINE-1 sequences is also considered potentially responsible for tumorigenesis [67].

## 3. Physiological Functions of TEs in the Host Cells

**Figure 2:**
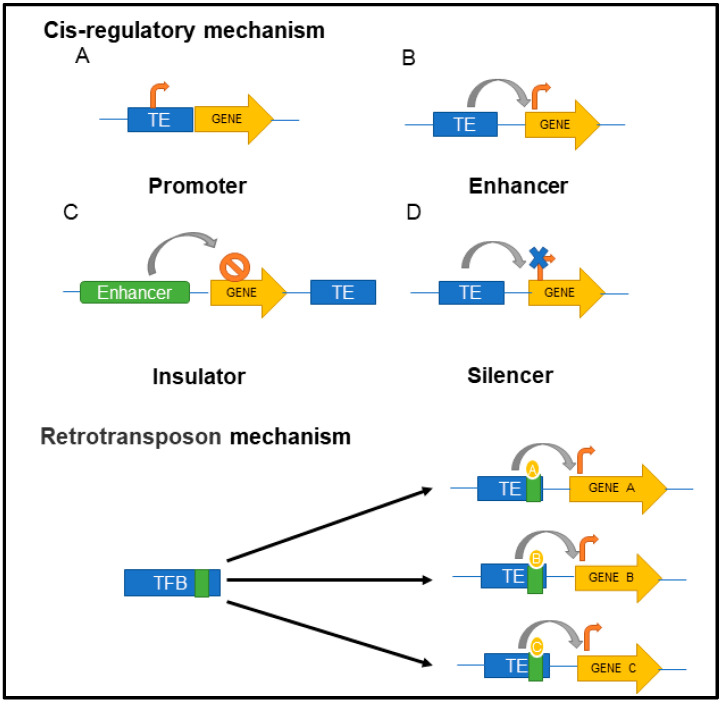
TE-regulated mechanisms of action in the host cells. (1) Cis-regulatory mechanisms involving (**A**) promoter and (**B**) enhancer, integrate the activity of specific transcription factor; (**C**) insulator, act either through enhancer-blocking activity or chromatin barrier activity; (**D**) silencer, silence the expression of genes. (2) Retrotransposon mechanism can increase the potential of transcription binding factor. (orange arrowhead indicates increased activity, blue cross indicates silencing of activity, circle with single cross indicates insulation of gene activity, grey arrow indicates direction of action).

Several studies have suggested that TEs are involved in critical physiological functions such as regulation of stem cell properties, epithelial to mesenchymal transition, inflammation, and many more biological functions [40]. Evidence also suggests that TEs in mammals have contributed to adaptive characteristics such as increased gene expression, gene replication, and stress tolerance [68]. Of note, some of the most important physiological contributions are from the TE-derived genes, which in turn participate in a variety of biological functions [40]. One such example is the *Metnase/SETMAR* gene, which is a fusion gene comprising histone H3 methylase gene and a transposase belonging to the mariner transposon family; it has been shown to function as a domesticated primate transposon playing critical roles in non-homologous end-joining of DNA repair and replication [69] and confers resistance to ionizing radiations [70]. Transposable elements are crucial components in tissue development and morphology [71] as well as impact in synaptic plasticity and cognition [72].Transposon element expression is associated with a cytokine response and induces recruitment and infiltration of immune cells in cancer and other inflammatory diseases. Based on their mechanistic functions, TEs in mammals are categorized into TE-containing functional RNAs and TE-containing cis-regulatory elements [40] (Figure 2). In the former category, TE-derived microRNAs (miRNAs) and long noncoding RNAs (lncRNAs) modulate function of protein-coding genes. For example, LINE-2-derived miRNA target sites have been identified in the 3′UTR of several genes, including housekeeping genes, thus resulting in LINE-2-mediated posttranscriptional regulation of these genes [73] Likewise, TE sequences found in the lncRNAs (~80% of lncRNAs) exert their function either by constituting a functional domain of the lncRNA or by regulating the host transcripts by changing their expression, localization, or processing [74]. The cis-regulatory elements in TEs, on the other hand, modulate gene expression by acting as epigenetic modifiers, transcription factor binding sites, or insulator binding proteins [40]. Human endogenous retroviruses have been associated with both activation and downregulation of the host immune system, besides their role in genomic instability and trans-regulation of human genes [75].

## 4. Regulation of TEs in the Host Cell

**Figure 3:**
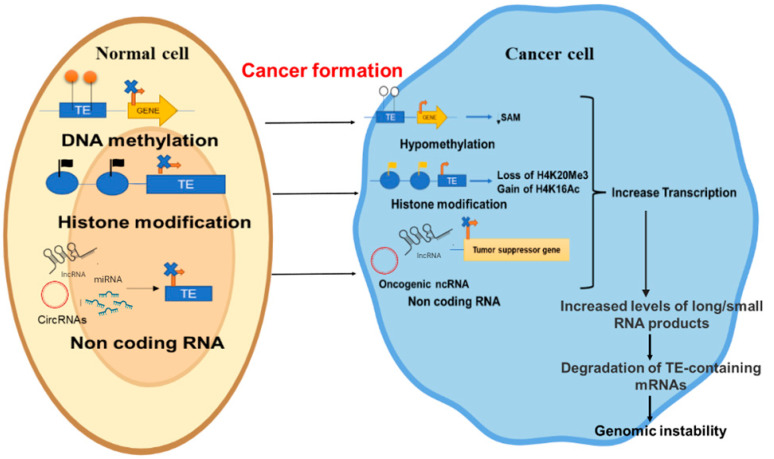
Regulation of TEs in the normal and cancer cell. In normal cells (**left panel**), epigenetic modifications like DNA methylation, histone modification, and non-coding RNA, silence the activity of TEs. In cancer cells (**right panel**) hypomethylation, different histone modification, and non-coding RNAs cause removal of repressive signals and unregulated expression of TEs. This leads to degradation of DNA, mutations, and genomic instability (orange arrowhead indicates the increased activity, blue circle indicates histone, orange circle indicates methyl groups, blue cross indicates silencing).

It is evident that TEs pose a potential threat to genomic stability and normal functioning of a cell. However, host cells have evolved several mechanisms to restrict the TE-mediated adverse effects, including DNA methylation [76], heterochromatin formation [77], histone modifications [78], and mRNA editing [76], which are often found lost in the cancer cells (Figure 3). Among these, DNA methylation is responsible for silencing most of the TEs in the somatic cells [79].

### 4.1. DNA Methylation

DNA methylation is a regulatory epigenetic process that works by recruiting repressor proteins, leading to transcriptional repression [80]. DNA methylation in mammalian genomes is catalyzed by DNA methyltransferases (DNMTs), which catalyze the transfer of a methyl group from S-adenyl methionine (SAM) onto C5 cytosine to form 5-methylcytosine [46]. The dynamics of DNA methylation are changed during development, which includes both demethylation and de novo methylation. De novo methylation is catalyzed by DNMT3A and DNMT3B, which establish methylation marks onto unmethylated DNA templates [81], whereas DNMT1 copies methylation marks from parental DNA onto newly synthesized DNA during replication and maintains constitutive methylation patterns [82,83]. DNA methylation acts as a repressor of the disruptive potential effects of TEs. However, during early developmental stages, DNA is demethylated globally, thereby releasing TEs to insert into germline and potentially pose a threat to genomic integrity [84]. Erasure of DNA methylation in developmental animal models leads to increased expression and propagation of TEs throughout the host genome [85,86]. Given the potentially adverse impacts TE expression can cause, Bestor [87] proposed that the silencing of TEs mediated through cytosine methylation allows the TEs to coexist with the host genome. This had led to the hypothesis that DNA methylation is a result of an evolutionary process that renders the sequence inactive in order to reduce the total amount of DNA available for DNA-binding regulatory proteins [87]. This hypothesis was confirmed by a recent study showing that TE insertion and its subsequent methylation upon insertion not only causes genome expansion, but the TE DNA methylation extends to the flanking regions of the host DNA [41]. The impact of TE methylation is not confined to the silencing, but it was believed and later confirmed by Zhou et al. that the increased transition rate of methylated cytosine to thymine in the TE DNA and other mutations confer a new cis-regulatory function to the TEs [41].

### 4.2. Histone Modifications

Epigenetic regulation is a crosstalk between DNA methylation and posttranslational histone modifications that include acetylation, methylation, ubiquitylation, and others [88]. In the absence of DNA methylation, the H3K9me3 andH3K27me3 epigenetic marks take over the regulation of a large spectrum of transposons to maintain genomic stability [89]. DNA methylation is linked to histone posttranslational modifications, which are affected by a ubiquitin-like PHD- and RING-finger domain containing protein (UHRF1), which in association with H3K9me2/3, is responsible for DNA methylation maintenance [90]. Since the genome has evolved through retrotransposon insertions, eventually host cells have developed ways to silence retrotransposon activity [91]. It has been reported that in primates, retrotransposon repression is achieved by KRAB-associated protein-1 (KAP1) recruited by KRAB zinc finger (KZNF) proteins, proteins that rapidly coevolved with retrotransposons to inhibit active retrotransposons like LINE-1 and SVA elements [91]. Further, KZNF/KAP1 proteins were also reported to work in association with DNMT3A/DNMT3B to maintain DNA methylation at imprinting control regions during early embryogenesis [92].

### 4.3. Silencing of Retroelements in Germ Cells

Germ cells and early embryos undergo epigenetic reprograming events that modify repressive DNA methylation and histone modifications [93]. In this scenario, small-RNA-mediated pathways are directed to regulate retrotransposon expression. RNAi limits the expression of repetitive parasitic sequences of murine endogenous retrovirus-L (MuERV-L) and intracisternal A particle (IAP), thus ensuring genomic integrity during developmental stages [94]. Also, discovery of Piwi proteins and their associated piRNA identified these proteins as regulators of retrotransposons via widespread mechanisms [95]. The mechanism of defense mediated by piRNAs against retrotransposon invasion is highly conserved among animal species [96]. The piwi piRNA pathway works as a transcriptional silencing machinery, as evidenced in the translocation of piRNA along with mouse piwi 2 (MIWI2) to the nucleus of progenitor germ cells [97]. Mouse knockout models of the Piwi proteins miwi-like (MILI) and MIWI2 showed de-repression of retrotransposons, thus indicating a role for these proteins in retrotransposon repression [98]. High throughput screening studies of MILI and MIWI2 suggest that piRNAs are not essential for the maintenance of de novo methylation [99,100]. However, later it was reported that the Piwi–piRNA complex is required for de novo DNA methylation of retrotransposon sequences and is associated with repressive chromatin remodeling activities [101,102]. Piwi-interacting RNA binds to Piwi proteins and guides the binding to target RNAs of expressed retroelements through sequence complementarity and further cleavage of the target RNAs to repress the reverse transcription of retrotransposons. Secondary piRNA biogenesis pathways also lead to cleavage of sense and antisense strands of retrotransposons that are mediated by piwi–piRNA complex where piRNA is target-sequence-derived [99]. Thus, the cumulative effects of DNA methylation, histone modifications, and RNA-mediated pathways play a significant role in recognizing and silencing retrotransposon activity in germline cells. The host cell in turn is dependent upon these retroelement regulatory pathways to preserve genomic integrity.

### 4.4. Silencing of TEs by miRNA and Other Mechanisms

Both in developing and mature mammalian cells, DNA methylation plays a crucial role in silencing different classes of TEs. However, the role of miRNA and several proteins in silencing TEs have also been reported. Endogenously produced miRNAs show a similar function as siRNAs and participate in the regulation of normal cellular processes, including TE-silencing. Depletion of DNMT1 counteracts transposon activation in murine embryonic stem cells (ESCs) via acute depletion of Ago2 or Dicer [103]. Inhibition of Dicer or Ago2 expression revealed that small RNAs are involved in an immediate response to demethylation-induced transposon activation, while the deposition of repressive histone markers follows as a chronic response [103]. Hamdorf et al. [104] reported that miR-128 acts as a direct repressor of LINE-1 retrotransposition in pluripotent stem cells and human cancer cells. In human-induced pluripotent stem cells and male mouse germ cells, piRNAs restrict LINE-1 retrotransposition [104,105]. However, another mechanism has been identified involving the tripartite motif 5α (TRIM5α) cytoplasmic retroviral sensor and limiting LINE-1 retrotransposition through recognition of the LINE-1 ribonucleoprotein complex [106]. Through its interaction with LINE-1 ribonucleoproteins, TRIM5α efficiently represses human LINE-1 TEs by restriction and by activation of the transcription factors AP-1 and NF-kB, which cause down-regulation of LINE-1 promoter activity [106]. These results are supported by the presence of ERVs in other human cancers with increased levels of TE expression [107,108].

Another study in murine ESCs demonstrated that, alpha-thalassemia/mental retardation X-linked (ATRX) and death-associated protein (DAXX) recruited the H3K9 histone methyltransferase SUV39H and mediated TE repression [109]. Moreover, LINE-1 silencing in primary murine embryonic fibroblasts occurs via chromatin remodelers such as SWI/SNF2-related, matrix associated, actin-dependent regulator of chromatin, subfamily A, member 6 (SMARCA6) [110]. We have also shown that E2F/RB1 mediates LINE-1 silencing in murine embryo fibroblasts [111]. Also, ERV silencing in murine ESCs occurs in an ATP-dependent manner through SWI/SNF-like remodeler SMARCAD1 and plays a critical role in resection as well as stress response [111,112,113]. Overall, histone modifications can silence TEs through a complex array of methods using several enzyme complexes. Using the Bari1 element as a model, it was demonstrated that the mobility of TEs can be effectively repressed by catalytically inactive polypeptides encoded by the transposase gene, showing existence of yet another mechanism of TE silencing [114]; however, it is not known if a similar mechanism exists in human cells as well, considering that Bari1 elements are absent in human genome.

## 5. TEs Associated with Other Diseases

Transposable elements, especially retrotransposons are known to contribute to several diseases other than cancer [8,10,35,115] (Table 1). Here we will briefly discuss the various mechanisms through which TEs cause diseases and refer the readers to a recent review for further reading [116]. It is reported that among the genetic disorders caused by TE insertions, X-linked disorders caused by LINE-1 insertions are abundant. However, the mechanisms behind TE insertion and X-linked disorders remains yet to be fully explained [117]. Significant progress has been made in understanding the different mechanisms through which TEs exert their deleterious impact on the cellular function as well as genomic stability, resulting in the manifestation of a disease. One mechanism involves TE expression; expression of TEs generally is induced through loss of TE promoter silencing either autonomously or through epigenetic alterations. This can lead to the production of TE-encoded proteins, RNAs analogous to retrotransposition intermediates, chimeric transcripts, lncRNAs, or altered gene regulation [118,119]. Altered TE expression is frequently observed in different cancer but has also been observed in neurological diseases, autoimmune and inflammatory disorders, and normal aging [116]. Another mechanism involves TE insertions, a highly deleterious process responsible for several monogenic diseases. This can happen either through the integration of TEs into an exon, thereby changing gene transcript, or into an intron, thereby altering mRNA splicing [116]. Several genetic disorders have been associated with this mechanism, such as Dent’s disease, Duchenne muscular dystrophy, neurofibromatosis type 1, hemophilia A, and many more [22]. Insertion of TEs can also introduce new patterns of mRNA splicing due to the presence of cryptic splicing sites in the inserts, which in some cases is just a single splice site and in other cases a pair of sites. Some examples of diseases associated with this cryptic splicing are Lynch syndrome (OMIM 614337), retinoblastoma (OMIM 180200), chronic granulomatous disease (OMIM 306400).

Several studies have reported that TEs in central nervous system (CNS) can be closely associated with brain disorders [15,135]. Nevertheless, the investigation and evidence of these illnesses in preclinical models is relatively limited. Stress is the main contributing factor to neuropsychiatric diseases, and evidence has been mounting on the relationship between TEs and stress-related disorders, including the roles of maternal behavior, stress, and exercise on rates of transposition in the murine hippocampus [136]. One recent study demonstrated that the N6 methyladenine modification is upregulated in the prefrontal cortex of mice due to stress, with about half of the modification being targeted to LINEs, resulting in the downregulation of RNA expression encoded by these elements [137].

Acute exposure of rats to stress downregulates Intracisternal A-particle (IAP)-LTR and B2 SINE in the hippocampus, while IAP-LTR and LINE-1 were upregulated in the cerebellum. Moreover, global increases in the H3K9me3 repressive histone marker in the hippocampus were observed at retrotransposon-containing loci [138]. Cappucci and colleagues also showed that repeated stress after 5 days altered LINE-1 expression [139] with evidence also supporting that under normal conditions, in the rat hippocampus a subclass of LINEs are transcribed, thus identifying a non-stress physiological role of TEs as well. The role of HERV-W in schizophrenia is also know as it was reported that in recent-onset schizophrenia patients there is a high HERV reverse transcriptase activity and that overexpression of HERV-W env in U251 glioma cells alters dopamine D3 receptor (DRD3) and brain neurotrophic derived factor (BDNF) levels, both known to increase the risk for schizophrenia [139]. Another study demonstrated that dysregulation of Alu expression mechanically leads to macular damage in rodent models and human systems [140]. Subsequent studies reported that siRNA-induced SINE RNA silencing leads to Dicer-1 expression downregulation which in turn causes degeneration through the NOD-, LRR-, and PYD-containing 3 (NLRP3) inflammasome pathway. This evidence suggests that TEs are associated with various brain-related physiological and pathological pathways [141]. For instance, migraine and epilepsy are linked to cortical spreading depression (CSD), with a rodent model study showing that CSD is connected with H3K9me3 LINE-1 modifications and changes in DNA methylation in the cortical genome [142,143]. In neurons and cultured fibroblasts, HERV-W is activated by T. gondii and cytomegalovirus infections [143,144].

In neurodegenerative disorders, TEs also have been shown to exert a crucial role. Transposable element activation through TAR DNA binding protein 43 (TDP-43)-regulated siRNA-silencing in a frontotemporal region causes dementia and amyotrophic lateral sclerosis (ALS) in *Drosophila* model [145,146], as well as in chick and human cell [147]. Ramesh et al. showed that knockdown of the *UHRF1* gene in the cerebellum of developing mouse brain upregulates murine IAP endogenous retrovirus, resulting in intense neurodegeneration [148]. On the other hand, the neurotoxic effect of mercury has been associated with increased LINE-1 expression in human neuroblastoma cell lines [149].

Cumulative reported preclinical studies in different models suggest the physiological significance of TEs in genetic disorders, including cancers and CNS-associated neuropsychiatric diseases (Table 2). However, in-depth research is needed to develop solid preclinical models to understand TE-driven diseases and to develop novel therapeutic approaches to manage these conditions.

## 6. Futuristic Therapeutics Involving TEs

TEs can alter gene expression and production of protein, making these elements suitable targets for drug development. The jumping nature of TEs has made them a useful tool in insertional mutagenesis procedure [160] with a potential application in bio-pharmacology and gene therapy [161,162]. Because of their non-viral origin, DNA transposons are considered safe for therapeutic applications [163]. Several preclinical studies have been conducted in different animal models of human diseases to test the feasibility of TEs as gene therapy tools (Table 2).For the treatment of many acquired and inherited human diseases, gene therapy using TE-based vectors has indeed proven to be a promising strategy. By exploiting properties such as its integration capacity and non-viral nature, many DNA-transposon-based vectors were adapted for gene therapy protocols. The Sleeping Beauty (SB) transposon, a member of the Tc1/mariner superfamily and first developed by Ivics and colleagues [164], has proven to be a successful approach for generating antitumoral T lymphocytes by ex-vivo transfer of TCR/CAR genes [165,166]. Furthermore, these T cells showed stable expression and anti-tumor activity in preclinical trials [167,168]. SB transposon system also proved to be efficient in achieving stable expression in modified human CD34+ hematopoietic stem cells [169]. A study showed the functional activity of piggyBac transposons by modifying human T cells that were capable of eliminating CD19, expressing human lymphoma cell lines [170]. Studies have indicated that apart from DNA transposons, retrotransposons, and particularly LINE-1, have become a potential treatment strategy for various diseases due to their various properties. One study demonstrated that antisense promoter LINE-1 demethylation by DNMT1 inhibitors is associated with increased transcription of neighboring genes [171]. This study implicated a critical role of TEs in epigenetic therapy, considering their side effects and therapeutic impact. Human ERV-W-Env expression in type 1 diabetes is an interesting example of therapeutic antibody use. The abnormal expression of ERV-Env protein in circulation and postmortem acinar pancreatic cells exemplifies a pathological cause in T1D patients revealed by epidemiological studies [172]. Recently, GNbAC1 monoclonal antibody (IgG4) directed against the HERV envelope protein was shown to specifically neutralize the human ERV-W-Env via inhibiting release of TNF-α in vivo and in vitro. Thus, IgG4 may be developed as a treatment for human ERV associated complex diseases. For instance, treatment with 18 mg/kg GNbAC1resulted in significant remyelination in multiple sclerosis (MS) patients [173], which may in near future lead to the development of mAb-based treatment for MS.

In preclinical and clinical studies, the epigenetic activation of immune cells leads to immune signaling in cancer. Treatment with DNMT inhibitors (DNMTis) in mouse models sensitizes melanoma cells to subsequent anti-CTLA4 therapy. Additionally, it also enhances anti-PD-1 therapy in murine ovarian cancer [174,175]. In a mouse model of ovarian cancer, DNMT inhibition plus histone deacetylase (HDAC) inhibition caused the activation of CD8+ T-cells through interferon signaling [176]. Another report in a xenograft mouse model of colorectal cancer treated with the DNMTi azacytidine (Aza) and dsRNA-destabilizing enzyme adenosine deaminase acting on RNA (ADAR1) showed synergistic anti-tumor action [177]. The ERV-K promotes intracellular fusion of breast cancer cells to endothelial cells, resulting in escape of malignant cells from the breast tissue and metastasis [177,178]. Similarly, in a syngeneic mouse model, ERV-K in renal cells induced the development of pulmonary metastases [179].

One of the enzymatic activities encoded by ORF2 in LINE-1 elements is a target for RT inhibitors. In fact, RT inhibition has been studied as a target in prostate cancer. Using RT inhibitory drugs or inhibition through RNA interference-dependent signaling of active LINE-1 reduces proliferation of cancer cells and mediates differentiation. Evidence has been presented that these drugs also cease the tumor progression in rodent models [180,181].

*p53* is a tumor-suppressor gene that is active in somatic cells and plays a vital role in double-stranded DNA break (DSB) repairs. In more than 50% of cancers, *p53* is mutated, with malignant cells deficient in p53 showing increased levels of TE activity, which further promotes genome instability and alterations [182]. The role of *p53* in repressing repetitive elements is an important safeguard against genomic instability induced by repetitive elements. Recently it was demonstrated that activation of p53 by MDM2 inhibitor induces expression of ERVs through inhibition of lysine-specific histone demethylase 1(LSD1) and DNMT1, which in turn trigger expression of type I/III interferons and promote T-cell infiltration [183]. The authors further demonstrated that inhibition of MDM2 in patients with melanoma activates viral mimicry and tumor inflammation genes [183], thus providing evidence for EVR expression-mediated cancer treatment. One study reported that activated synthesis of ORF2 protein following transcriptional activation of LINE-1 halts double-stranded breaks in chromatin and upregulates p53 activity [184]. The cells might undergo apoptosis regulated by a feedback mechanism responding to DNA damage. Haoudi and colleagues support this proposed mechanism, showing that LINE-1 upregulation induces apoptosis in the G418R HCT wild-type *p53* cell line [185]. However, this did not occur in the mutant *p53* G418R SW480 cell line, signifying the therapeutic potential of LINE-1 inhibitors for wild-type *p53* malignancies [185,186]. The fanconi anemia factor and DSB repair restrict LINE-1 retrotransposition during the S/G2 phase [187], implicating LINE-1 retrotransposition in the pathogenesis of ovarian and breast cancers lacking in DSB protein BRCA1, BRCA2, an E3 ubiquitin ligase with a key role in several DNA repair pathways. These results suggest the existence of a ‘battleground’ at the DNA replication fork between homologous recombination (HR) factors and L1 retrotransposons and reveal a potential role for L1 in the genotypic evolution of tumors characterized by BRCA1 and HR repair deficiencies [187].

In cancer therapy, cancer cells treated with DNMTi activate the type I and III interferon signaling pathways and enhance the immune cascade of tumors triggered by the detection of double-stranded RNA (dsRNA) derived from TEs [188,189,190]. Azacytidine (Aza) and 5-aza-2′-deoxycytidine (Dac) are two promising DNMTis approved by FDA for the treatment of hematological malignancies. Aza binds covalently to the DNMT1 preventing it from methylating the DNA, thus turning on tumor suppressing genes. In cell lines derived from lung, breast, ovarian, and colon cancers, low doses of azacytidine (Aza) and 5-aza-2′-deoxycytidine (Dac) boost the immune signaling involving cytokines, antigen processing, and interferon responses [191,192]. Additionally, Aza, in a series of kinase reactions, gets converted into Aza triphosphate, which is then incorporated into RNA, thus altering RNA metabolism and protein synthesis, causing cytotoxicity [193]. Ribonucleotide reductase reduces Aza diphosphate into Dac diphosphate, which is phosphorylated to triphosphate and merged into DNA, altering DNA methyl transfer activity and leading to hypomethylation of replicating DNA [193]. DNMTi decreases total DNA methylation, and as a result, dsRNA increases; specifically, dsRNA achieved from inverted-repeat Alu elements [177]. The sensors in cytoplasm TLR3 and MDA5 sense this change in genetic material and activate the canonical interferon signaling pathways under viral mimicry [194]. Studies also revealed that DNMTi treatment-induced interferon response was antagonized by inhibiting the dsRNA sensors TLR3 and MDA5, showing that the transcription of TE-induced dsRNA species activates the interferon response [189,190]. Additional studies have reported that DNMTi treatment induced viral mimicry; further increases have been found upon treatment with HDAC inhibitors (HDACi), Vitamin C, or inhibition of H3K9 methyltransferase [194,195].

Cell-mediated immunity and humoral immunity have been studied in the context of TEs. Antibodies against ERV-K have been identified in several cancers, including ovarian and melanoma patients and teratocarcinoma cell lines [196]. In cancer patients, T-cell-mediated and autologous humoral response were reported to aid in the development of adaptive immune activity to target TEs as novel therapeutic targets. Transposable elements may be potential therapeutic targets in various complex diseases, including genetic disorders such as cancers and CNS related disorders. Transposable elements can be used to insert or delete sequences at will, thus allowing for targeted manipulation of gene expression and alterations in pathophysiological pathways. Identification of specific TEs in the pathogenesis of the disease may allow for development of specific oligonucleotide sequences targeting these sequences.

Transposon elements are overexpressed in the majority of human diseases due to demethylation of the TE loci [51]. However, in a few cases, the TE transcript process is independent of the DNA methylation [86]. This suggests another mechanism of TE that controls non-coding RNAs and histone modifications. Modification of RNA has an important role in human diseases [197]. One of the modifications of transposon RNA M(6)A, has been reported [198]. Such modalities where transposons are involved in human diseases may be potential targets for therapeutics. For further research, in addition to RNA modifications, one might investigate the uncharacterized DNA modifications of TEs. One example is m6dA, which is present in the human genome at specific sites, is associated with increased transcription activity, and has been involved in cancer [199,200]. In the future, investigating the correlation between TE loci and markers upregulated in disease would be an interesting research area.

## 7. Clinical Trial

Many clinical trials are currently targeting TEs or taking advantage of TE biology (Table 3). Recently, combining anti-CTLA-4 treatment with DNMTi in melanoma has given promising results in terms of anti-tumor activity and improvement in immune activation [201]. Although no direct correlation between the treatment and TEs was deduced from the study, it is plausible that the TE pathway is affected by the inhibition of DNMT, as shown previously [177,188,189,190]. Several studies have revealed that inhibition of CDK4/6 leads to suppression of DNMT1 due to hypomethylation of TEs, specifically in ERVs, and this can further be used to induce viral mimicry [202,203,204]. Additionally, treatments in some on-going clinical trials may involve TE activity due to the targeted pathways. For instance, the phase Ib NIBIT-M4 clinical trial testing the next-generation DNA hypomethylation agent guadecitabine in combination with ipilimumab showed improved immunomodulatory and anti-tumor activity in advanced melanoma [201], which may involve perturbation of the epigenetic regulation of TEs and more importantly, the ERVs and their subsequent immunomodulatory effect.

Currently, checkpoint inhibitor therapy is being used for immune signaling in clinical trials for kidney, ovarian, colorectal, and melanoma cancers (NCT01928576, NCT02811497, NCT02546986, NCT02530463, NCT02961101, NCT03019003, NCT02397720) involving the TE signaling pathways. In a recently published clinical trial data for colorectal, breast, and ovarian solid tumors, where durvalumab and T-cell survival promoter (PD-1 antagonizing monoclonal antibody) combined with oral form of hypomethylation agent azacytidine CC-486 were investigated in an open-label phase II METADUR trial (NCT02811497), investigators failed to detect a strong DNA demethylation associated with the lack of the expected viral mimicry inflammatory response [205]. These studies, however, have shown the importance of making stable and good pharmacokinetic epigenetic therapies to get promising results in the treatment of solid tumors.

The results of early phase I/II trials (NCT03389035) in B cell acute lymphoblastic leukemia (B-ALL) were recently published. In this study, nine adult and four pediatric patients were given infusion doses of Chimeric antigen receptor (CAR) T cells. CAR T cells were made with Sleeping Beauty (SB) transposons to convert the cells into cytokine-induced killer (CIK) cells. The result showed complete remission and a durable response for up to 10 months [206]. Another on-going clinical trial is performing TE control pathway analysis in tumor samples collected from the surgical parts and their comparison to normal adjacent tissues. Clinical trial NCT02171845 is studying the various factors and mechanisms of TE suppression during fetal gonad development; however, the status of this study is currently unknown. A B-cell lymphoma phase I trial (NCT04289220) using anti-CD19 CAR T-cells engineered using the PiggyBac transposons in an invitro cell line derived xenograft model showed vigorous tumor-killing activity with no obvious side effect related to the cytokine storm and neurotoxicity.

In a single center phase1/2 study, a Hurler syndrome patient underwent allogeneic hematopoietic stem cell transplantation treated with engineered autologous plasma blasts to show α-L-iduronidase (IDUA) by using the SB-transposon system (NCT04284254). In pediatric and adult patients with r/r BCP-ALL open-label, multi-center, phase II study, the therapeutic efficacy and safety of PTG-CARCIK-CD19 cell infusion (NCT05252403) will be determined. The PTG-CARCIK-CD-19 infusion consists of genetically modified allogeneic T lymphocytes in suspension. In a phase II trial (NCT04102436) with metastatic cancer patients, SB engineered transposons/transposase autologous T-cells administration showed T-cell receptor expression reactive against neoantigens [162,163]. Another engineered EGFR-CAR T-cell through non-viral piggyBac system therapy in non-small cell lung cancer (NSCLC) patients was revealed to be feasible and safe (NCT03182816) [164]. Collectively, these studies emphasize the importance of understanding TE biology so that this knowledge can be exploited for the development of novel direct and indirect strategies to combat cancer.

## 8. Future Perspectives

One of the most important revelations to emerge from human genome project is that TEs are widely represented in the genome, underscoring their biological importance in shaping our genome and their potential roles in regulating the function of the host cell. Indeed, this has been revealed through numerous studies showing that TEs are not merely “JUNK DNA”, but rather, are genetic elements that play a crucial role in numerous biological processes, including regulation of gene expression, genome evolution, genetic variability, and cancer initiation and progression, to list a few. The insertion and deletion of TEs in the genome and their epigenetic reactivation can lead to genetic disorders, some mild and some severe. Transposon elements have gained increasing recognition as major regulators of genetic function.

**Figure 4:**
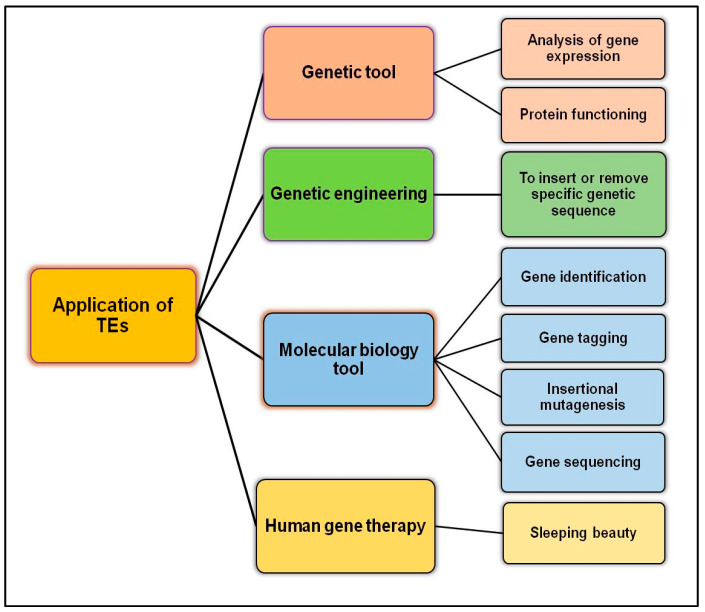
Potential applications of TEs in biological science and human health.

Advancements in techniques and their development, such as next-generation sequencing and different computer-based applications, will help researchers and the medical community understand the role of TEs in human disease. The healthcare and scientific community already recognize the importance of individualized therapy for genetic disorders such as cancer. All this progress and development may soon lead to the development of promising TE-based novel therapeutic approaches for the treatment of many illnesses. For instance, the role of DNMTi in causing ‘viral mimicry’ through the expression of TEs, as discussed earlier, is an important advancement in the treatment of several diseases, especially cancer. Some recent studies, although not directly linked to TEs, have showed that cyclin-dependent kinases (CDKs) are necessary for type I interferon production; the inactivity among CDK4/6 may be sufficient to counter the post-transcriptional block of INFβ through CKD1/2/4 inhibitor R547 [207,208]. Cyclin-dependent kinase 4/6 inhibitors (CKD4/6i) selectively target proliferating cancer cells and can be an excellent therapeutic strategy to enhance or boost the immune signaling in cancer cells without hampering normal cells. Another future application of TEs is in the recombinant DNA technology (rDT), as it is estimated that the global rDT market will reach 850 billion USD by 2025, of which TE-based expression vectors will be of a substantial proportion [209]. Besides these, application of TEs in basic as well as applied biological sciences is in offing (Figure 4), transforming “foes into friends”.

## Figures and Tables

**Table 1:** Transposable elements and their associated human diseases.

Type of TE	Genetic Disorder/Disease	References
LINE-1 promotor hypomethylation.	Lung, Colon, Pancreatic, Ovarian Cancer	[120]
LINE-1 insertion in exon 14 of factor VIII gene	Hemophilia A	[11]
LINE-1 insertion	Familial Retinoblastoma	[22]
LINE-1 insertion in 3′noncoding region of fukutin gene	Fukuyama type congenital muscular dystrophy	[121]
LINE-1 insertion in DMD gene	Duchene muscular dystrophy	[122]
LINE-1 intronic insertion in RP2 gene	Retinis pigmentosa	[123]
LINE-1 insertion	Coffin-Lowry Syndrome	[124]
LINE-1 insertion in PDHX gene	Pyruvate dehydrogenase complex deficiency.	[125]
Alu insertion in exon 1 of CD40LG gene	Higm Syndrome	[126]
Alu insertion in CLCN5 gene	Dent’s Disease	[127]
Alu intronic insertion in NF1 gene	Neurofibromatosis type1	[18]
Alu insertions	Colon, Breast, Ovarian Cancer	[20,128,129]
Alu insertion in APC gene	Leukemia	[130]
Alu insertion in QAT gene	OAT deficiency	[131]
Alu insertion in COL4A3 gene	Alport Syndrome	[132]
SVA insertion in exon 6 of factor VIII gene	Hemophilia B	[133]
SVA insertion in intron 7 of PMS gene	Lynch syndrome	[134]

**Table 2:** Preclinical studies on transposon-based gene therapy.

Sr. No.	Transposon	Animal Model	Delivery	Disease	References
1.	Sleeping Beauty Transposons	Dogs	Liver-Directed Hydrodynamic Delivery		[150]
2.	Sleeping Beauty Transposons	Dogs	Liver-Directed Delivery		[151]
3.	Retrotransposon activation in Alzheimer’s disease	Mouse		Alzheimer’s disease	[152]
4.	PiggyBac Transposons	Mice		Duchenne Muscular Dystrophy	[153]
5.	Sleeping Beauty (SB)	C57BL/6 J mice	Hydrodynamic Tail Vein Injection	Hepatocellular Carcinoma	[154]
6.	Transposon-triggered innate immune response confers cancer resistance	blind mole rat		Cancer	[155]
7.	Corticosterone dynamically regulates retrotransposable element expression	Rat		Stress condition	[156]
8.	Differential Responses of LINE-1	Rat		Psychomotor impairments	[157]
9.	Spatially Resolved Expression of Transposable Elements	Mice		Neurodegenerative disease amyotrophic lateral sclerosis	[158]
10.	Activation of HERV-K(HML-2)	Human pluripotent stem cells		Disrupts cortical patterning and neuronal differentiation	[159]

**Table 3:** Clinical advances in transposon-based gene therapy.

Sr. No.	Study	Disease	Intervention/ Treatment	Phase	Clinical Trial Gov. Identifier
1.	MT2018-18: Sleeping Beauty Transposon-Engineered Plasmablasts for Hurler Syndrome Post Allo HSCT	Mucopolysaccharidosis Type IH (MPS IH, Hurler Syndrome), Mucopolysaccharidosis Type IH MPS IH, Hurler Syndrome	Autologous Plasmablasts	1/2	NCT04284254
2.	Analysis of Transposon Control Pathways in Germinal Cancers of the Testicle	Germinal Cancers of the Testicle	Genetic: Extraction of total RNA from healthy and tumor tissues		NCT02873793
3.	Transposon-manipulated Allogeneic CARCIK-CD19 Cells in Paediatric and Adult Patients With r/r ALL Post HSCT (CARCIK)	Acute Lymphoblastic Leukemia in Relapse	Biological: CARCIK-CD19	1/2	NCT03389035
4.	Anti-CD19 CAR in PiggyBac Transposon-Engineered T Cells for Relapsed/Refractory B-cell Lymphoma or B-cell Acute Lymphoblastic Leukaemia	B Cell Lymphoma, B-cell Acute Lymphoblastic Leukemia	Biological: Anti-CD19 CAR-T Cells Injection	1	NCT04289220
5.	Mechanisms and Factors Responsible for the Inhibition of Transposons During Fatal Gonad Development in Humans	Medical Termination of Pregnancy, Voluntary Termination of Pregnancy	Other: surgical biopsies		NCT02171845
6.	Measurable Residual Disease Driven Strategy for One or Two Infusions of Non- Viral, Transposon-manipulated CARCIK (CD19) Cells: A Phase II Study in Paediatric and Adult Patients with Relapsed/Refractory B Cell Precursor ALL (BCP-ALL)	Acute Lymphoblastic Leukemia	Genetic: PTG-CARCIK-CD19	2	NCT05252403
7.	A Phase II Study Using the Administration of Autologous T-Cells Engineered Using the Sleeping Beauty Transposon/Transposase System to Express T-Cell Receptors Reactive Against Mutated Neoantigens in Patients with Metastatic Cancer	Endocrine/ Neuroendocrine, Non-Small Cell Lung Cancer, Breast Cancer, Gastrointestinal/ Genitourinary Cancers, Ovarian Cancer	Drug: Fludarabine Drug: Cyclophosphamide Drug: Aldesleukin Biological: Sleeping Beauty Transposed PBL	2	NCT04102436
8.	Phase I/II Study of Autologous T Cells Engineered Using the Sleeping Beauty System to Express T-Cell Receptors (TCRs) Reactive Against Cancer-specific Mutations in Subjects with Solid Tumors	Gynecologic Cancer, Colorectal Cancer, Pancreatic Cancer, Non-small Cell Lung Cancer, Cholangiocarcinoma, Ovarian Cancer	Biological: Neoantigen specific TCR-T cell drug product Biological: Aldesleukin (IL-2)	1/2	NCT05194735

## Data Availability

Not applicable.
